# Enhancing our understanding of ways to analyze metagenomes

**DOI:** 10.3389/fgene.2014.00108

**Published:** 2014-04-29

**Authors:** Frank Emmert-Streib

**Affiliations:** Computational Biology and Machine Learning Laboratory, Faculty of Medicine, Health and Life Sciences, Center for Cancer Research and Cell Biology, School of Medicine, Dentistry and Biomedical Sciences, Queen's University BelfastBelfast, UK

**Keywords:** meta genomics, genomics, multivariate analysis, microbial organisms, statistical analysis

Metagenomics is a relatively new field that applies modern genomics techniques to study communities of microbial organisms directly in their natural environments (Chen and Pachter, 2005; Tringe et al., 2005). In this way, it avoids the need for isolation and lab cultivation of individual species that provided a major obstacle of cultivation-based methods. For this reason, the field offers enormous opportunities to enhance our understanding of the microbial world in general with potential applications in many different areas, e.g., ecology, agriculture, biotechnology and medicine (Gill et al., 2006; Cox-Foster et al., 2007; Chistoserdova, 2010; Virgin and Todd, 2011).

Unfortunately, due to the novelty of the field, designing statistical analysis methods and guiding procedures for goal oriented analysis of such data sets are still at its infancy. The paper by Dinsdale et al. (2013) aims to fill this gap by providing a numerical comparison of a variety of different clustering (K-means, unsupervised random forest and partitioning around medoid), classification (linear discriminant analysis, classification tree, Random Forest, canonical discriminant analysis), dimension reduction (principal component analysis and canonical discriminant analysis) and visualization methods (multidimensional scaling) for metagnomics by studying the metabolic functions of 212 microbial metagenomes within and between 10 environments. For this reason, the data set used for the numerical analysis was grouped into 10 different environments (coastal marine water, deep water, saline evaporation pond, mat community, open water, coral reef water, hydrothermal spring water, human associated, terrestrial animal associated, freshwater) and most environments were covered by multiple sequencing technologies.

Using this real data set allowed a discussion of the results of the individual analysis methods in a comparative manner revealing their advantages and disadvantages in a practical context. For instance, all analyses methods found the presence of phage genes within the microbial community to be a good predictor to classify a microbial community as “host-associated” or “free-living.” In addition to the comparative analysis, the paper explains also the used methods in a way that the reader does not need to be familiar with them before reading the paper. This makes the paper a comprehensive, introductory source of information. Overall, this is a very helpful study for scientists interested in metagenomics, particularly microbial ecologists, to understand how the methods behave for a real data set making this paper much more useful than generic review papers.

On a statistical note, the paper by Dinsdale et al. (2013) covers methods from three important areas of machine learning and statistics (Clarke et al., 2009; Haste et al., 2009). First, unsupervised learning methods to analyze data without a label are covered by discussing a variety of clustering methods. Second, supervised learning methods to analyze data with a label, e.g., a class identifier to distinguish different environments from each other, are included for some of the most important classification methods. Third, visualization methods are forming a natural starting point for any statistical data analysis in general and for an exploratory data analysis (EDA) (Tukey, 1977) in particular. For this reason, it is very important to add visualization methods to the paper for reminding the reader that a data visualization should always be part of a metagenomcis analysis because it can help for getting insights into such multivariate and high-dimensional data.

There are a couple of additional topics I would like to have seen included in the paper that are of relevance for metagenomics. First of all, for any multivariate data set there is the problem of a multiple testing correction (Dudoit and van der Laan, 2007) that needs to be conducted when testing statistical hypothesis. It would be interesting to know if metagenomcis data have characteristics that *deviate* from other genomics data, especially with respect to their covariance structure, or if similar procedures can be applied and which of these are recommended. Second, for classification and clustering methods it is necessary to perform a feature selection in a way that the actual analysis is conducted for lower dimensional profile vectors. For instance, a method like the *lasso* (Least Absolute Shrinkage and Selection Operator) (Tibshirani, 1994) that does not convolute covariates into meta-variables, e.g., like principle component analysis (PCA), but conserves the interpretation of the selected variables in terms of the original variables. This has the advantage that 'interesting' features correspond to well-defined *individual* genomic variables making a biological interpretation of obtained results usually easier. Third, it would have been interesting to discuss network-based systems biology approaches that are directly aiming to estimate interaction patterns between the covariates of the data (de Matos Simoes and Emmert-Streib, 2012). This would also allow to connect to visualization methods because the resulting network structures could be explored visually and in this way could lead to the generation of novel biological hypotheses about the problem.

Finally, I think it is also noteworthy to mention that the authors make the data and the R-code they used for their analysis publicly available (http://dinsdalelab.sdsu.edu/metag.stats/index.html) allowing the interested reader to reproduce the results of the paper. This is commendable and forms a good example for other studies. For ensuring the future availability of this supplementary information I suggest to deposit these files in the databases CRAN or Bioconductor.

## Conflict of interest statement

The author declares that the research was conducted in the absence of any commercial or financial relationships that could be construed as a potential conflict of interest.
